# Spatial variation and associated factors of unmet need for family planning among reproductive age women in Ethiopia, insights from 2021 PMA-Ethiopia data: multilevel logistic regression analysis

**DOI:** 10.1186/s12889-025-21917-y

**Published:** 2025-02-20

**Authors:** Abiyu Abadi Tareke, Eyob Ketema Bogale, Tadele Fentabel Anagaw, Misganaw Guadie Tiruneh, Eneyew Talie Fenta, Destaw Endeshaw, Habitu Birhan Eshetu, Amare Mebrat Delie, Ousman Adal Tegegne

**Affiliations:** 1 Amref Health in Africa, COVID-19 Vaccine/EPI Technical Assistant at West Gondar Zonal Health Department, Gondar, Ethiopia; 2 Health Promotion and Behavioral Science Department, College of Medicine and Health Science, Bahir Dar, Ethiopia; 3 Department of Health Promotion and Behavioral Science, School of Public Health, College of Medicine and Health Science, Bahir Dar University, Bahir Dar, Ethiopia; 4 Department of Health Systems and Policy, Institute of Public Health, College of Medicine and Health Sciences, University of Gondar, Gondar, Ethiopia; 5 Department of Public Health College of Medicine and Health Science, Injibara University, Injibara, Ethiopia; 6 Department of Adult Health Nursing, School of Health Sciences, College of Medicine and Health Sciences, Bahir Dar University, Bahir Dar, Ethiopia; 7 Curtin School of Population Health, Curtin University, Perth, WA Australia; 8 Department of Public Health, College of Medicine and Health Science, Injibara University, Injibara, Ethiopia; 9 Department of emergency, Bahir Dar University, College of Medicine and Health Sciences, Bahir Dar, Ethiopia

**Keywords:** Prevalence, Factors associated, Unmet need, Family planning, Spatial analysis, PMA, Ethiopia

## Abstract

**Background:**

The unmet need for family planning (FP) occurs when women want to limit or delay childbearing but lack access to contraception, leading to unintended pregnancies and increased maternal and child mortality, especially in developing countries. This study uses the 2021 PMA-Ethiopia survey to assess unmet need for family planning (FP) among reproductive age women, addressing limitations in previous research that relied on outdated and non-representative data. The findings offer valuable insights to help policymakers address local challenges and improve reproductive health outcomes in Ethiopia.

**Methods:**

This study utilized secondary data from the Performance Monitoring for Action Ethiopia survey conducted in 2021. A total of 5,203 reproductive-aged women were included in this study. STATA version 16 was used to cross-tabulate and fit the models. To account for the hierarchical structure of the data, we employed multilevel logistic regression. We estimated four statistical models: a null model (Model 0) to assess between-community variations, Model I incorporating individual-level predictors, Model II adding community-level factors, and Model III including both. To select the best-fitting model, we conducted model comparison using BIC, AIC, deviance, and log-likelihood ratio (LLR) to assess model performance. We calculated adjusted odds ratios along with their corresponding 95% confidence intervals (CIs). Furthermore, a significance level of *p < *0.05 was considered as strong evidence of statistical significance.

**Results:**

The prevalence of unmet need for family planning among reproductive-age women was 23.60% [95% CI: 22.46%, 24.78%]. Model comparison indicated that Model III exhibited the best fit, with the lowest AIC (5306) and DIC (5300) values, along with an improved log-likelihood (-2650). Factors positively associated with unmet need included women aged 45–49 [AOR = 4.7, 95% CI: (2.97, 7.43)], 40–44 [AOR = 3.21, 95% CI: (2.23, 4.61)], 35–39 [AOR = 2.47, 95% CI: (1.78, 3.44)], and 30–34 years [AOR = 2.42, 95% CI: (1.76, 3.33)] compared to those aged 15–19 years. Conversely, having 1–2 children [AOR = 0.47, 95% CI: 0.39, 0.56], having 3–4 children [AOR = 0.50, 95% CI: 0.39, 0.63], and having five or more children [AOR = 0.51, 95% CI: 0.39, 0.66] were factors negatively associated with unmet need compared to women who are para 0. Furthermore, the spatial pattern of unmet need for family planning exhibited clustering (Moran’s index = 0.25, *p-*value = 0.0039). The primary cluster is located in the central part of Oromia, northern SNNP, northwest of Sidama, and northeastern part of Southwest Ethiopia.

**Conclusion:**

This study affirms the ongoing high prevalence of unmet need for family planning in Ethiopia. It identified women’s age and parity as crucial factors correlated with unmet need for FP. Additionally, there exists an unequal distribution of the burden of unmet need for FP across the country. To address the varying reproductive health needs at different life stages and parity levels, the interventional plan needs to be based on women’s age and the number of children they have. Moreover, location-tailored interventional plans should be employed to optimize family planning related service inequalities.

## Introduction

The World Health Organization (WHO) promotes universal access and human rights for family planning (FP) and sexual and reproductive health services [1]. Modern FP programs offer many benefits, such as enabling women to safeguard against unwanted pregnancies [2], reducing complications related to unintended pregnancies, enhancing education and economic opportunities, contributing to birth spacing [3], empowering women [4] and narrowing gap of gender inequalities [1]. Ethiopia, characterized by a total fertility rate (TFR) of 4.6, is categorized among high fertility nations globally [5]. Furthermore, the country exhibits a notable population growth rate, while facing a remarkably high unmet need for FP [6] and low prevalence rate of contraceptive usage [5], which stands at 15% [5]. Addressing the gap in contraceptive usage and enhancing modern FP methods can contribute to better health outcomes for mothers and children.

Unmet need for FP is a phrase used to describe women who have an apparent demand for contraception services either to limit the desired number of children or delay childbearing but unable to use any modern contraception method [7, 8]. Unmet need FP is an important public health concern that can cause negative sequelae for both individuals and communities. It is a pillar indicator for monitoring/evaluating the progress of FP programs and an indication of the success of reproductive health programs. It is one of the main reproductive health indicators in the sustainable development goal (SDG indicator 3.7.1) mentioned as “the Proportion of women who have their need for FP satisfied with modern methods” [9]. The unmet need for FP is a reflection of the gap in access to health care services [10].

In Africa, especially in Ethiopia, Eswatini, Guinea-Bissau, Madagascar, Malawi, Rwanda, Uganda, and Zambia unmet need for FP is highest when compared to another continent [11]. Women who have an unmet need for FP are at high risk of experiencing unwanted or untimed pregnancies which lead to negative outcomes like maternal and maternal mortality [12, 13], unsafe abortion [14] and economic divide.

Regional comparisons play a vital role in advancing global efforts, such as the SDGs, by enabling a better understanding of progress, challenges, and opportunities in different areas.

To address the existing gaps in previous studies, this research strives to provide comprehensive insights into the notion of the unmet need for FP among reproductive age women in Ethiopia. The study aims to unveil the most recent performance in terms of reproductive health, with a specific focus on FP. This previous studies [15, 16] used survey, being seven years old (the 2016 Ethiopian demographic health survey), inherently fails to capture the dynamic changes and evolving challenges that Ethiopia’s reproductive health sector may have undergone in the intervening period. But this study aims to generate more accurate information related to unmet need for FP using more recent and representative data i.e., 2021 PMA-Ethiopia survey, to overcome the limitations posed by the outdated 2016 EDHS.

One of the key challenges encountered in previous studies conducted in Ethiopia is the utilization of non-representative data. To tackle this, our study integrates national representative data obtained from the 2021 PMA-Ethiopia survey [17]. This approach ensures that the findings reflect the most recent developments in the field of reproductive health concerning the unmet need for FP. The comprehensive analysis of the most recent data allows for a robust evaluation of the situation across the country.

To enhance the statistical power and obtain accurate estimations, this research employs an advanced statistical model called multilevel logistic analysis. By utilizing this model, the hierarchical nature of the data is properly accounted for, resulting in more precise findings. This analytical approach further strengthens the reliability of the study and contributes to a better understanding of the factors associated with unmet need for FP among reproductive age women in Ethiopia.

Unlike previous studies in Ethiopia, this research goes beyond simply assessing the national-level scenario and explores the spatial pattern of unmet need for FP. By employing a potent tool called SaTScanTM software, this study evaluates sub-regional estimates, shedding light on local (geographic) variations. This spatial modeling approach is particularly beneficial for local decision makers, as it equips them with valuable information needed to make immediate and informed decisions regarding FP services.

Therefore, this study plays a vital role in unmasking the health services inequalities related to FP in Ethiopia. By using nationally representative data from the 2021 PMA-Ethiopia survey and advanced statistical techniques, our research offers a comprehensive assessment of the spatial variations and factors associated with unmet need for FP. The findings provide valuable insights for policymakers to effectively address the unique needs of different regions in Ethiopia. This study aims to make a meaningful contribution to improving reproductive health outcomes in the country.

## Methods and materials

### Study design and period

#### Study area

**Fig. 1:**
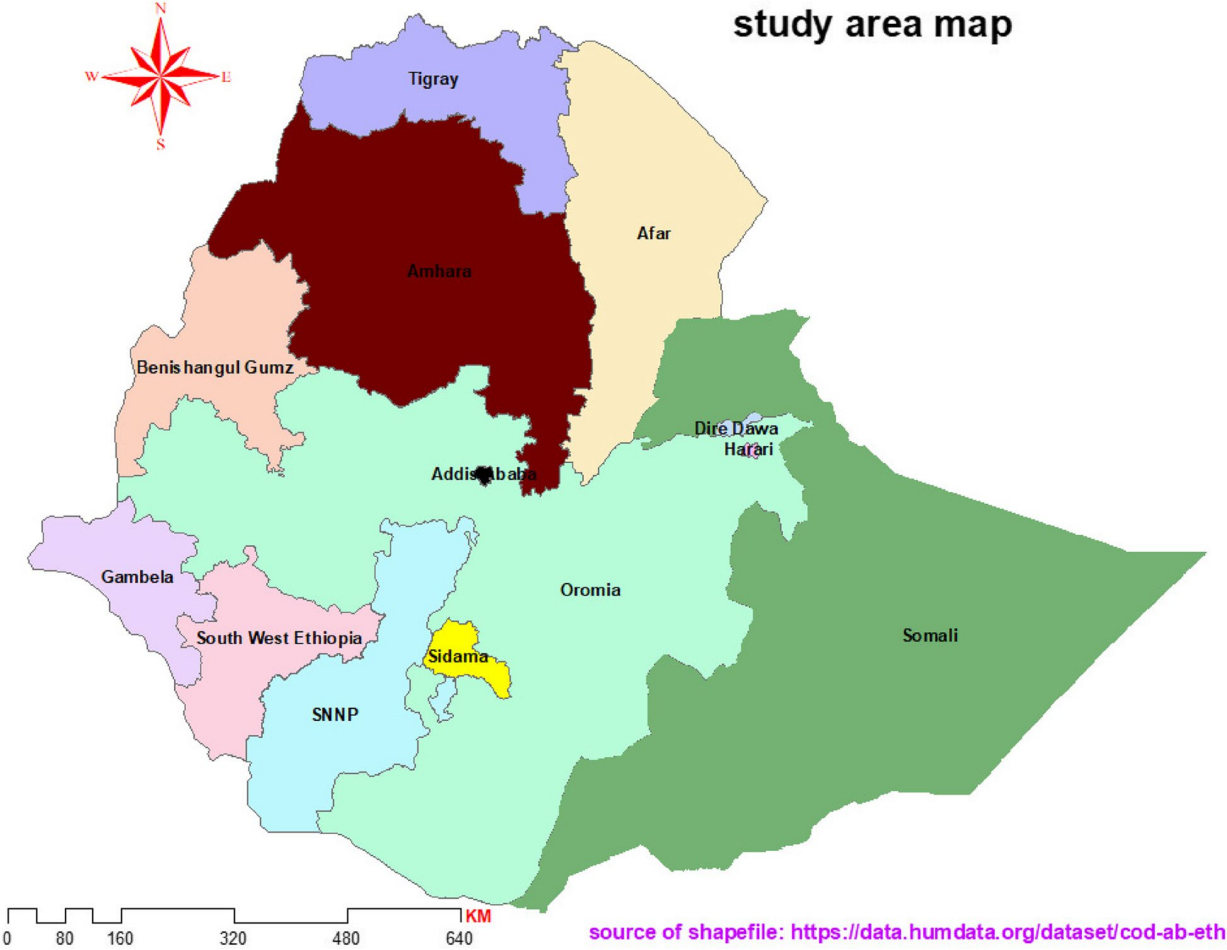
Administrative region Ethiopia in 2021

Ethiopia, the second most populous nation in Africa, has a tiered administrative structure that goes from regions to Kebeles. The 2007 Census showed that 83.6% of the population lived in rural areas, with an average household consisting of 4.7 people. Additionally, 47% of women were in the 15 to 49 age range [18]. The country comprises 11 regions, including Tigray, Oromia, Amhara, and SNNP, as well as two chartered cities: Addis Ababa and Dire-Dawa (Fig. 1). These regions are further divided into zones, woredas, and finally Kebeles.

#### Population

##### Source population

All reproductive age women (15–49 years) in Ethiopia.

##### Study population

Women aged 15 to 49 years who are eligible to use modern FP methods but are not infertile, pregnant, or experiencing amenorrhea during the data collection period.

#### Data sources

Data for this study was derived from PMA Ethiopia survey which was carried out in October and November 2021. The survey was conducted in collaboration between Addis Ababa University and Johns Hopkins Bloomberg School of Public Health. It is composed of three different survey components: an annual cross-sectional survey which is conducted nationally, a panel survey that follows pregnant women through one year postpartum, and an annual Service Delivery Point (SDP) survey. This study used the data generated from the first (cross-sectional) survey.

#### Sampling procedure

The study utilized a nationally representative sample derived from the 2021 PMA-Ethiopia survey, which employed a two-stage stratified cluster sampling design. In the first stage, 243 enumeration areas (EAs) were randomly selected from the Central Statistical Agency’s master frame, stratified by Ethiopia’s 11 administrative regions and urban/rural classifications. In the second stage, 35 households per EA were randomly selected, yielding a final analytic sample of 5,203 eligible reproductive-aged women (15–49 years). Data were weighted to account for the complex survey design and ensure representativeness.

### Study variables

#### Dependent variable

The dependent variable is divided into two categories: “unmet” and “met.”

Unmet need for FP: refers to the proportion of fertile, sexually active women in a union who lack access to modern contraceptive methods for spacing or limiting births and would prefer to do so [19]. Those women using modern FP methods are classified as having met their FP needs. Pregnant women, those unable to conceive, women who have reached menopause, and individuals not currently sexually active are excluded from the study.

#### Independent variables

We categorized the independent variables into two distinct groups: level-1 factors, which pertain to the individual level, and level-2 factors, which pertain to the community level.

For level-1 factors, we considered women’s age, women’s educational level, marital status, final FP decision-maker, wealth quintile, parity, and place of residency.

As for level-2 factors, we identified community-level variables, namely, community level of poverty and community level of illiteracy. These community-level factors were derived by aggregating individual-level factors at the cluster/community level.

#### Spatial analyses

To explore, create, visualize and edit the spatial information of unmet need for FP, we employed ArcGIS version 10.8 software. For assessing the spatial pattern of unmet need for FP, we utilized an inferential statistic known as the Spatial Autocorrelation (Global Moran’s I) tool [20]. This tool calculates Moran’s I Index value along with a z-score and *p-*value to determine whether the pattern of unmet need for FP is scattered, clustered, or random. The significance of Moran’s Index is evaluated based on both the z-score and *p-*value (Table 1). The unit of spatial analysis in this study is the enumeration areas (EAs) or clusters, which were selected from the master sample frame, rather than individual respondents’ GPS locations or regions.

**Table 1:** *P-*value interpretations and their implications for unmet need analysis

The *p-*value is not statistically significant	You cannot reject the null hypothesis. It is quite possible that the spatial distribution of unmet need is the result of random spatial processes
The *p-*value is statistically significant, and the z-score is positive	You may reject the null hypothesis. The spatial distribution of high values and/or low values in unmet need is more spatially clustered
The *p-*value is statistically significant, and the z-score is negative	You may reject the null hypothesis. The spatial distribution of high values and low values in unmet need is more spatially dispersed

We conducted a purely spatial analysis using SaTScan™ version 10.0.1 software to identify statistically significant spatial clusters (geographic areas with a high number of women having unmet need for FP), which enabled us to pinpoint regions that warrant targeted interventions. The analysis employed the Bernoulli model-based purely spatial scan statistic with 1/0 event data, where cases (1) represent ‘unmet’ and controls (0) represent ‘met’ FP needs. We retained the default maximum spatial cluster size setting (50% of the population at risk). This setting is recommended as it allows identification of both small and large sized clusters without pre-selection bias regarding cluster size.

#### Multilevel logistic regression analysis

STATA version 16 software was utilized to perform cross-tabulations and compute summary statistics. The PMA datasets exhibit a hierarchical structure, with individuals nested within sample clusters (specifically, enumeration areas). This structure leads to observation dependencies and violates the equal variance assumption in single-level statistical models such as standard logistic regression [21]. The equal variance assumption posits that residual variance should be consistent across all levels of the independent variable. However, in this hierarchical data structure, such consistency is not achievable.

The PMA survey employs a multistage stratified clustered sampling approach, where women within a cluster are more likely to share similar traits compared to those between clusters. To address these challenges, we employed a multilevel logistic model to identify individual and cluster-level factors that collectively influence unmet FP needs.

We began by calibrating a random intercept model (null model). This method is valuable for establishing the nesting of observations within clusters and justifying the use of multilevel analysis by examining the intra-class coefficient (ICC). The ICC reveals the extent of similarity among study subjects within a cluster and the degree of variation across clusters.

To compare and select models, we utilized Akaike information criterion (AIC), deviance information criterion (DIC), Log-likelihood Ratio (LLR), and Bayesian information criterion (BIC). The model exhibiting the lowest values for these parameters was chosen as the best-fit model.

The study conducted a comparison of four models: a null model without independent variables, Model I incorporating only individual-level factors, Model II including only community-level factors, and Model III combining both individual- and community-level independent variables. In the multivariable mixed-effect logistic regression analysis, variables with a *p-*value below 0.2 in the bi-variable analysis were included. Significant factors associated with unmet need were identified using Adjusted Odds Ratios (AOR) with a 95% Confidence Interval (CI) and *p-*value ≤ 0.05 in the multivariable model.

#### Ethical consideration

This study used PMA-Ethiopia datasets obtained through the online database (https://datalab.pmadata.org) with authorization, exempting it from the need for ethical approval.

## Results

### Characteristics of the study population

Most of the study participants were from Oromia (39.79%), Amhara (22.98%), and Benishangul-Gumuz (18.99%). The median age of women was 28 years [inter-quartile range: 23–35]. Most of the women 3,643 (70%) were residing in rural and more than half of them were primary level educated.

The study population is predominantly married (90.84%), with only small percentages living with a partner (2.82%), divorced or separated (2.67%), widowed (0.69%), or never married (2.97%), while the distribution of wealth statuses shows a relatively balanced demographic, with 20.18% in the lowest category, 19.89% in the lower, 19.52% in the middle, 19.23% in the higher, and 21.18% in the highest wealth category (Table 2).

**Table 2:** Characteristics of the study population (*n* = 5203)

Characteristics	Weighted frequency	Percent
Region of residency		
Tigray	305	5.86%
Afar	54	1.04%
Amhara	1195	22.98%
Oromia	2070	39.79%
Somali	195	3.75%
Benishangul-Gumuz	57	1.09%
SNNP	988	18.99%
Gambella	20	0.39%
Harari	22	0.43%
Addis Ababa	274	5.26%
Dire Dawa	23	0.44%
**Maternal age**		
15–19	457	8.79%
20–24	1027	19.75%
25–29	1323	25.44%
30–34	913	17.55%
35–39	839	16.13%
40–44	479	9.20%
45–49	164	3.15%
**Marital status**		
Married	4726	90.84%
Living with a partner	146	2.82%
Divorced / separated	139	2.67%
Widow / widower	36	0.69%
Never married	155	2.97%
**Wealth status**		
Lowest	1,050	20.18%
Lower	1,035	19.89%
Middle	1,016	19.52%
Higher	1,001	19.23%
Highest	1,102	21.18%
**Final FP decision maker**		
You alone	1,153	22.17%
Provider alone	101	1.93%
Partner alone	115	2.22%
You & provider	114	2.20%
You & partner	725	13.93%
Missing values	2,994	57.55%
**Parity**		
None	2,526	48.55%
1–2	1,551	29.81%
3–4	622	11.95%
5 +	504	9.69%
**Place of residency**		
Rural	3,643	70.03%
Urban	1,559	29.97%
**Educational status**		
Never attended	2263	43.45%
Primary	1880	36.13%
Secondary	648	12.46%
Technical & vocational	192	3.68%
Higher	221	4.24%

### Spatial distribution of unmet need for FP

**Fig. 2:**
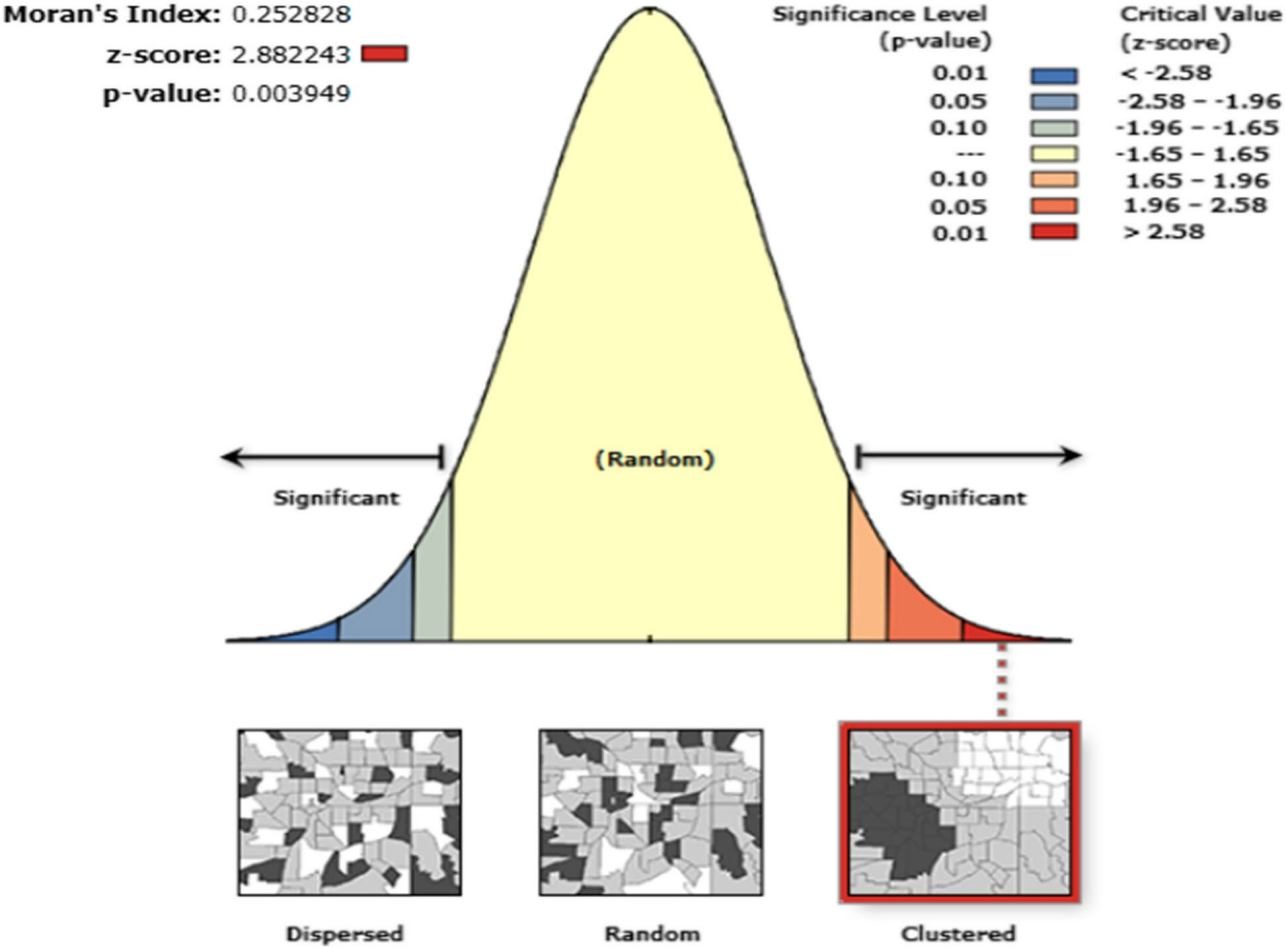
Spatial Autocorrelation (distribution) of unmet need for FP in Ethiopia, 2021

From Fig. 2 we can understand that the spatial pattern of unmet need for FP in Ethiopia is moderately clustered (Moran’s index = 0.25 and *p-*value = 0.003949). Having a positive Moran’s index with a z-score value of 2.88 is interpreted as the likelihood of the clustering pattern of unmet need for FP due to chance is less than 1% (Fig. 2). Having non-random spatial patterns justifies the further search for hotspot areas (areas having a high number of women having an unmet need for FP).

### Spatial clustering of unmet need for FP

A total of Fifty-seven statistically significant clusters of unmet need for family planning were identified and categorized into primary and secondary clusters. Among these, 34 were the most likely clusters, representing critical focal points for targeted interventions, while the remaining 23 clusters were classified as secondary clusters respectively (Table 3).

**Table 3:** Detailed cluster characteristics unmet need for FP in Ethiopia, 2021

Types of clusters	Coordinates/Radius	No. cluster	Cluster location	Expected cases	Observed cases	RR	*p-*value
Most likely cluster	(7.911053 N, 37.916754 E) / 143.60 km	34	Central part of Oromia, north SNNP, north-west of Sidma, north-east of south west Ethiopia	204	864	1.57	< 0.0001
Secondary cluster 1	(6.570971 N, 41.974927 E) / 288.52 km	12	Eastern Oromia, most of Somali region (especially southern part)	115	490	1.65	< 0.0001
Secondary cluster 2	(13.159408 N, 38.054771 E) / 54.65km	11	On the border of SNNP and Oromia	72	306	1.62	< 0.001

**Fig. 3:**
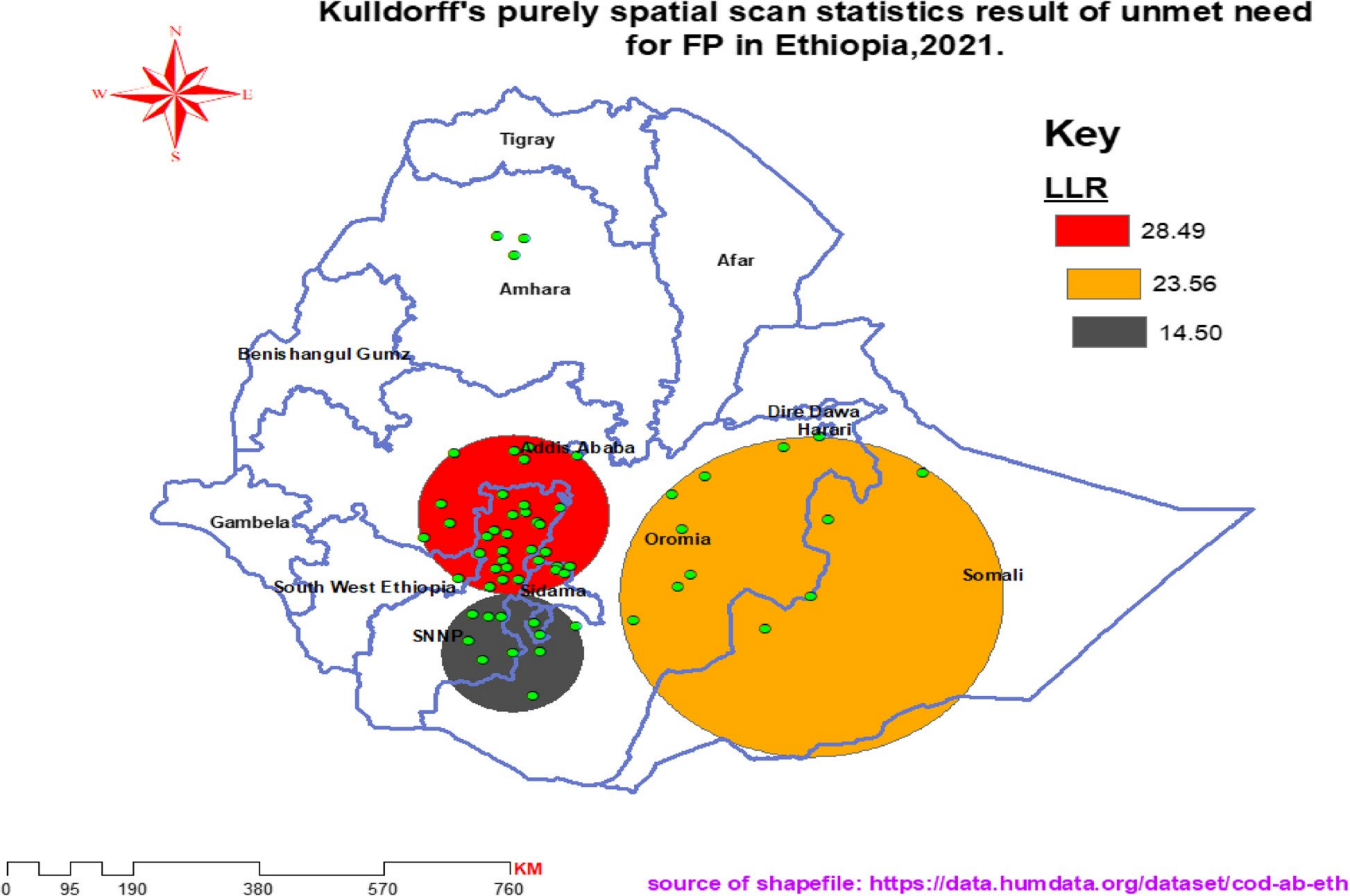
SaTScan analyses of statistically significant unmet need spatial clusters in Ethiopia, 2021

The primary clusters, with the highest likelihood ratio, were predominantly located in the northern part of the Southern Nations, Nationalities, and Peoples’ (SNNP) region and central Oromia region, centered at (7.911053 N, 37.916754 E) within a radius of 143.60 km (Fig. 3). Within these primary clusters, the probability of unmet need for FP was 57% higher compared to areas outside these clusters, as indicated by a relative risk of 1.57 (Table 3).

### Rate of unmet need for FP

**Fig. 4:**
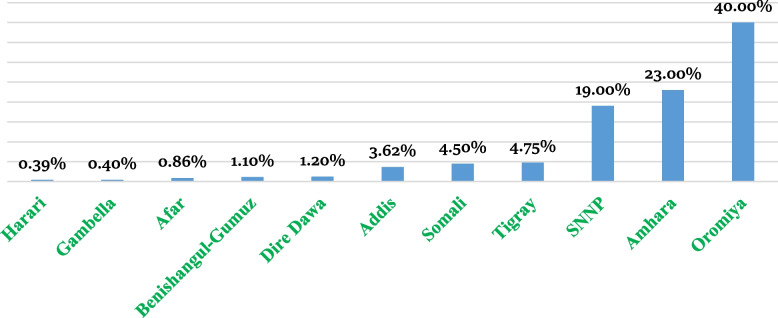
Rate of unmet need for FP in Ethiopia by administrative regions, 2021

The rate of unmet need among reproductive age women in Ethiopia was 23.60% [95% CI: 23.46, 24.78]. The highest prevalence of unmet need for FP is noticed in the Oromia region with a prevalence rate of 40% [95% CI: 38%, 41%] followed by Amhara region (23%) and SNNP (19%). Conversely, the lowest prevalence was also noticed in Gambella (0.4%), Harari (0.39%) and Benishangul-Gumuz (1.00%) regions (Fig. 4).

### Factors associated with unmet need

The Intraclass Correlation Coefficient (ICC) was computed to assess the intra-cluster variability among study participants. Women within the same cluster were more likely to exhibit similar characteristics than those from different clusters. The output from the intercept-only (null) model revealed that the ICC is 9.9%. This suggests that 9.9% of the variation in unmet need is attributed to differences between the clusters. The finding from ICC was also supplemented finding from median odds ratio (MOD).

The median odds ratio value of 1.77 in the null model (Table 4) indicates that when two individuals with identical characteristics (covariates) are randomly selected from different clusters, those from higher-risk clusters have a 77% higher likelihood of experiencing unmet need for FP compared to individuals from lower-risk clusters. The proportional change in variance from model III (the full model) shows that both individual and community-level factors account for 23% of the odds of unmet need for FP. Proportional change in variance from model III (full model) illustrates that, 23% of the odds of unmet need for FP was accounted by both individual and community level factors.

**Table 4:** Comparison of models and fitness parameter results

Fitness parameter	Null model	Model I	Model II	Model III
Community level variance	0.36	0.27	0.32	0.277
ICC	9.91% [7.34, 13.24]	7.75% [5.47, 10.87]	8.83% [6.45, 12.00]	7.77% [5.41, 10.91]
MOR	1.77[1.62,1.96]	1.64[1.51,1.82]	1.71[1.56–1.89]	1.65[1.52,1.84]
PCV (%)	Reference	25%	11.11%%	23%
**Model fitness**				
Log- likelihood ratio (LLR)	−2755	−2651	−2744	−2650
DIC(−2LLR)	5510	5302	5488	5300
AIC	5515	5339	5497	5306
BIC	5528	5463	5523	5413

Null Model is the empty model, baseline model without any determinant variable. Model I is adjusted for individual-level factors. Model II is adjusted for community-level factors. Model III is final model adjusted for both individual- and community-level factors

### Model comparison

Based on the table below, model III, which incorporates both individual and community-level factors, is the best-fitting model for this dataset. This is because model III (the full model) has the lowest BIC and AIC values (Table 4).

The variables “final FP decision maker” and “marital status” were not included in the multivariable model due to a high number of missing values and *p-*values exceeding 0.2 in the bivariable model, respectively.

From the multivariable multilevel modeling, variable age and women’s educational status showed significant positive associated with the unmet need for FP in Ethiopia. In addition to this parity is negatively was negatively associated with unmet need prevalence.

Compared to women aged 15–19 years, the likelihood of unmet need for FP increased progressively with age: 2.42-fold (95% CI: 1.76–3.33) among those aged 30–34, 2.47-fold (95% CI: 1.78–3.44) at 35–39, 3.21-fold (95% CI: 2.23–4.61) at 40–44, and 4.70-fold (95% CI: 2.97–7.43) at 45–49 years (Table 5).

**Table 5:** Multivariable multilevel logistic analysis result of unmet need among reproductive age women in Ethiopia, 2021

Characteristics	Model I (95%CI AOR)	Model II (95%CI AOR)	Model III ((95%CI AOR)
**Women age**			
15 – 19 years	1.00		1.00
20– 24 years	1.36[1.00,1.86]		1.37 [1.01, 1.86] *
25– 29 years	1.65[1.22,2.24]		1.65 [1.22, 2.24] **
30– 34 years	2.42[1.76, 3.33]		2.42 [1.76, 3.33] **
35– 39 years	2.47[1.78, 3.44]		2.47 [1.78, 3.44] **
40– 44 years	3.21[2.23, 4.62]		3.21 [2.23, 4.61] **
45– 49 years	4.70 [2.97, 7.43]		4.70 [2.97, 7.43] **
**Women’s education**			
University	1.00		1.00
Secondary	1.08 [0.74, 1.55]		1.08 [0.75, 1.56]
Primary	1.15 [0.81, 1.61]		1.15 [0.81, 1.62]
Not educated	1.43 [0.99, 2.05]		1.45 [1.00, 2.08]
**Parity**			
Para 0	1.00		1.00
Para 1-para 2	0.47 [0.39, 0.56]		0.47 [0.39, 0.56] **
Para 3-para 4	0.50 [0.39, 0.64]		0.50 [0.39, 0.63] **
Para 5 and above	0.51 [0.39, 0.66]		0.51 [0.39, 0.66] **
**Wealth Quintile**			
Lowest	1.00		1.00
Lower	1.13 [0.91, 1.40]		1.13 [0.91, 1.41]
Middle	0.87 [0.68, 1.09]		0.88 [0.69, 1.11]
Higher	0.95 [0.72, 1.25]		0.98 [0.72, 1.32]
Highest	0.71 [0.48, 1.04]		0.73 [0.48., 1.11]
**Place of residency**			
Rural	1.00		1.00
Urban	0.85[0.65, 1.16]		0.87[0.61, 1.22]
**Community level literacy**			
High		1.00	1.00
Low		0.80 [0.63, 1.03]	1.05[0.81, 1.37]
**Community level poverty**			
Low		1.00	1.00
High		0.69 [0.54, 0.89]	0.91[0.65, 1.55]
Constant	0.098[0.09, 0.11]	0.34 [0.30, 0.40]	0.18[0.11, 0.28]

*AOR* adjusted odds ratio

*Note*:*=*p*-value <0.01 & ≥0.001, and **=*p*-value<0.001

The odds of unmet need for FP are reduced in women who have experienced childbirth compared to women with no history of giving birth. More specifically, the likelihood of encountering unmet demand for FP is 53% [AOR = 0.47, 95% CI: 0.39, 0.56], 50% [AOR = 0.50, 95% CI: 0.39, 0.63], and 49% [AOR = 0.51, 95% CI: 0.39, 0.66] lower in women who were para 1–2, para 3–4 and para 5 + respectively compared to para 0.

## Discussion

This study assessed the prevalence of unmet need for FP to assess and understand the extend of the issue. The study goes beyond reporting prevalence of unmet need for FP by identifying factors associated with unmet need for FP using nationally representative survey data. Besides, this study included spatial analysis to identify hotspot areas i.e., locations with higher number of clustered women experiencing unmet need for FP and the spatial information could guide This information can guide both higher and local health authorities to develop more targeted and localized interventions.

According to this study, the prevalence of unmet need for FP in Ethiopia is 23.60% with a 95% confidence interval of [23.46, 24.78]. This finding is lower than studies done in eastern Ethiopia (33%) [22], Addis Ababa Ethiopia (31%) [23], India (38%) [24], and Angola (51.7%) [25]. However, this finding is higher than study done in Nigeria (12.7%) [26]. The dissimilarity observed may be attributed to variances in socio-demographic attributes, geographical location, sample size, and cultural differences regarding contraception.

Using SaTScan statistics, a cluster of women having unmet need (women who are closer together in space than would normally be expected) were mainly located in Eastern Oromia, central Oromia and the Sidama region. Another spatial analysis conducted using the 2016 Ethiopian Demographic Health Survey data yielded clusters that were nearly indistinguishable from this finding. The spatial analysis using demographic health survey identified statistically significant clusters of women having unmet need for FP in most of Somali region, Sidama zone (currently region), and central Oromia [15]. As repeated clustering of women having unmet need was continued in those areas, we recommend for the upcoming researchers to conduct deep studies to uncover the real reasons behind the clustering of cases in those areas. It would be valuable to consider additional factors behind, such as demographic, socioeconomic, and cultural variables, to better understand the underlying reasons for the observed clusters. Increasing the uptake of contraception could reduce the number of women not using FP in those hotspot areas. We strongly recommend local health authorities near those identified hotspot areas (clusters) take their own investigations and measurements to address the clustering of high number of women with FP demand is unsatisfied. We urge policy makers and program designers to implement more effective strategies and interventional activities tailored to the specific needs and context of each hotspot area. Evaluate the accessibility and availability of contraceptive services in the identified hotspot areas. In the event of any deficiencies, strive towards enhancing the dissemination of contraceptives and ensuring that healthcare establishments possess the necessary resources to deliver contraceptive services.

The prevalence of unmet need for FP is observed to be higher among older women as compared to younger women. This finding is in line with a study done in Nigeria [27] which revealed that the unmet need for limiting is two-fold higher among women of advanced age in comparison to their younger counterparts. This can be justified as older women have less desire to have more children and a lower likelihood of using modern contraception [28–30] even though they didn’t desire more children. Those groups of women may think of themselves as less prone to unintended pregnancy. Age women being older, having fewer living children, not having regular access to health services, having given birth in the past year and having the desire for more children were associated with a lower likelihood of using contraception. The less likelihood of unmet need for FP among older women is also revealed in another scoping study using 34 articles from Low- and Middle-Income Countries (LMICs) [31]. This might be due to older women any more FP methods, especially for spacing their number of children.

When compared with women without children, those with more than five children have 53% less unmet for FP. Additionally, women with three to four and with one to two children have 50% & 49% fewer unmet need for FP respectively compared to women with no child. Women having too many children are more likely to use FP because they think that they have already accomplished their mission of having the number of desired children and are less likely to have unmet need. A study done in Indonesia reported a positive relationship between the number of children and FP usage [28] which reported that as the number of children increases the likelihood of using contraception also increases i.e., less likely to encounter unmet need.

### Implications and future research

Our research has implications for the Ethiopian Ministry of Health, public health policymakers, and researchers working on reproductive health. The findings provide actionable insights to address unmet needs for FP at both clinical and population levels.

The findings of this study carry important clinical and public health implications:

Clinically, the high unmet need for FP among older women emphasizes the necessity for tailored counseling and service delivery that addresses their unique concerns. Additionally, the lower unmet need among women with more children suggests a need to ensure equitable access to FP services for those with fewer children, integrating these services into maternal and postnatal care. Strengthening health systems to improve the quality and accessibility of FP services, particularly in areas identified as hotspots, is critical for reducing the unmet need and ensuring that women across all demographics can achieve their reproductive health goals.

From a public health perspective, the spatial clustering of unmet need in certain regions underlines the importance of geographically targeted interventions along with resource allocation. Policymakers and program designers should focus on addressing the disparities in FP access by enhancing healthcare infrastructure and addressing socio-cultural barriers in these regions. Community education and behavior-change campaigns can be instrumental in increasing FP uptake and reducing misconceptions, especially in identified hotspots. Such efforts align with global goals, such as SDG 3.7, and contribute to broader improvements in maternal and child health outcomes, reducing unintended pregnancies and associated risks. Through targeted and equitable approaches, this study provides a roadmap for optimizing FP programs and addressing unmet needs effectively. Finally, future research would benefit from focusing on the underlying reasons behind clusters of unmet need for FP identified in this study. Investigating socio-economic, cultural, and healthcare access factors in these regions could generate valuable insights to inform more effective and localized interventions 45 = .

### Strengths and limitations of the study

In conclusion, this study reveals both strengths and limitations that can be identified. The main strength of this study is, that utilizing national representative data advances the statistical power of the study. The large sample size contributes to the reliability of the findings and supports the generalizability of the results to the broader population. The authors explored the clustering of women having unmet need using the emerging statistical analysis called spatial analysis in the context of health systems. Location-specific responses to health problems based on identified clusters could help in narrowing health inequalities. In relation to the cross-sectional aspect, PMA Ethiopia encounters analogous constraints to those observed in other cross-sectional surveys (chicken-and-egg problem). Therefore, Cross-sectional studies are observational, provide a snapshot of information at a point in a time and descriptive in nature, and cannot be used to determine the cause of something. Hence, we used data from a secondary survey, and missing other behavioral and cultural factors in this study could compromised model performance.

## Conclusion

The prevalence of unmet need for FP in Ethiopia is still a public health concern (high). This may hinder achieving SDG aimed at increasing the proportion of women who have their need for FP satisfied with modern methods. Developing a geographically targeted interventional plan to curve this clustering of women having unmet need for FP is a proactive approach. Geographically targeted interventions are often more cost-effective than providing services randomly. By concentrating resources in areas with the greatest need, the impact of interventions can be maximized, potentially leading to a more rapid reduction in unmet need. Scaling up of maternal health services like FP by the government and other concerned bodies should consider those old women and women having fewer children. Further research should be conducted that mainly focuses on exploring specific cultural factors influencing contraceptive utilization, conducting more in-depth analyses in hotspot areas, or examining changes in unmet need over time (spatio-temporal relationship) increases.

## Data Availability

No datasets were generated or analysed during the current study.

## Abbreviations

AIC: Akaike information criteria
AOR: Adjusted Odds Ratio
BIC: Bayesian information criteria
FP: Family planning
PMA: Performance Monitoring for Action
DIC: Deviance Information Criterion
ICC: Intra-Class Correlation
LLR: Log- Likelihood Ratio
MOR: Median Odds Ratio
PCV: Proportion of Variance Change
CI: Confidence interval
LLR: Log-Likelihood Ratio
AOR: Adjusted odds ratio
SDG: Sustainable Development Goals
EA: Enumeration areas
SNNP: South Nation and Nationalities People
KM: Kilometer
